# Loss of Par1b/MARK2 primes microglia during brain development and enhances their sensitivity to injury

**DOI:** 10.1186/s12974-018-1390-3

**Published:** 2019-01-17

**Authors:** Victoria L. DiBona, Wenxin Zhu, Mihir K. Shah, Aditi Rafalia, Hajer Ben Cheikh, David P. Crockett, Huaye Zhang

**Affiliations:** Department of Neuroscience and Cell Biology, Rutgers Robert Wood Johnson Medical School, Piscataway, NJ 08854 USA

**Keywords:** Par1b, MARK2, Microglia, Priming, Injury

## Abstract

**Background:**

Microglia, the resident immune cells of the brain, exhibit various morphologies that correlate with their functions under physiological and pathological conditions. In conditions such as aging and stress, microglia priming occurs, which leads to altered morphology and lower threshold for activation upon further insult. However, the molecular mechanisms that lead to microglia priming are unclear.

**Methods:**

To understand the role of Par1b/MARK2 in microglia, we first expressed shRNA targeting luciferase or Par1b/MARK2 in primary microglial cells and imaged the cells using fluorescent microscopy to analyze for morphological changes. A phagocytosis assay was then used to assess functional changes. We then moved in vivo and used a Par1b/MARK2 knockout mouse model to assess for changes in microglia density, morphology, and phagocytosis using immunohistochemistry, confocal imaging, and 3D image reconstruction. Next, we used two-photon in vivo imaging in live Par1b/MARK2 deficient mice to examine microglia dynamics. In addition, a controlled-cortical impact injury was performed on wild-type and Par1b/MARK2-deficient mice and microglial response was determined by confocal imaging. Finally, to help rule out non-cell autonomous effects, we analyzed apoptosis by confocal imaging, cytokine levels by multiplex ELISA, and blood-brain barrier permeability using Evans Blue assay.

**Results:**

Here, we show that loss of the cell polarity protein Par1b/MARK2 facilitates the activation of primary microglia in culture. We next found that microglia in Par1b/MARK2 deficient mice show increased density and a hypertrophic morphology. These morphological changes are accompanied with alterations in microglia functional responses including increased phagocytosis of neuronal particles early in development and decreased surveillance of the brain parenchyma, all reminiscent of a primed phenotype. Consistent with this, we found that microglia in Par1b/MARK2 deficient mice have a significantly lower threshold for activation upon injury.

**Conclusions:**

Together, our studies show that loss of Par1b/MARK2 switches microglia from a surveillant to a primed state during development, resulting in an increased neuroinflammatory response to insults.

**Electronic supplementary material:**

The online version of this article (10.1186/s12974-018-1390-3) contains supplementary material, which is available to authorized users.

## Background

The precision and coordination required for developing a healthy central nervous system (CNS) is remarkably complex. Surprisingly, one of the first groups of cells to take residence in the CNS is a sub-population of immune cells of the monocyte lineage called microglia [1]. Before neurons and other macroglia are born within the CNS, microglia migrate into the brain rudiment, where they will reside and self-proliferate throughout life [2, 3]. Historically, microglia were believed to function solely as immune cells, which are in a ramified and resting state under physiological conditions, and become amoeboid-shaped and active during times of illness or insult [2]. However, they are now known to play an active role in constructing the developing brain. For example, microglia play a key role in clearing excess neurons during early brain development [4–6]. More notably, microglia also help refine the neuronal network by assisting with neuronal survival and proliferation [7, 8], synapse pruning [9–11], and synapse formation and function [12–15]. This remarkable array of novel functions attributed to microglia has led to the concept that ramified microglia under physiological conditions are in a surveillant state rather than a resting state [1, 3]. Yet, the mechanisms regulating microglia throughout development and between their various non-immune and immune-activated roles are poorly understood.

The conversion of microglia between their surveillant state and reactive states is neither binary nor linear in nature. Rather, microglia can adopt different pathways of activation depending on the nature of the stimuli [3]. Importantly, various intermediate levels of activation exist. Stressors such as aging and maternal immune activation (MIA) can convert ramified microglia in their physiological surveillant state to a primed state [16]. Compared with ramified microglia under physiological conditions, primed microglia may show a hypertrophic phenotype with increased branching but shorter process lengths, or a de-ramified phenotype with fewer and shorter process [17, 18]. These primed microglia have a much lower threshold for activation, which contributes to the exaggerated response of the aging brain to injuries [17]. However, the molecular mechanisms regulating the priming of microglia remain unclear.

From a cell biological perspective, the conversion of microglia between different functional states involves complex changes in cell polarity. Surveillant microglia are highly ramified and polarized; while amoeboid, reactive microglia show reduced cellular polarization/compartmentalization. Yet, during an active response to insult, microglia extend highly polarized processes toward the injury site. Thus, proteins regulating cellular polarity are likely involved in this intricate process. The par proteins are key regulators of polarity establishment in many different cellular contexts. These proteins were discovered in the *C. elegans* zygote, where mutations in the *par* genes cause a defect in partitioning of the zygote into asymmetric daughter cells [19, 20]. One of the par proteins is the Ser/Thr kinase Par1, also known as microtubule affinity regulating kinase (MARK) [21]. There are four members of the Par1/MARK family in mammals. Par1/MARK plays an instrumental role in regulating various cellular processes in the brain including neuronal migration [22–24], neurite outgrowth [25], dendritic spine formation, and plasticity [26–31]. However, whether and how Par1 affects microglia function has yet to be explored.

In this study, we show microglia express the polarity protein Par1b/MARK2, and that deficiency of Par1b/MARK2 expression results in both morphological and functional activation of microglia. Primary microglia cultures depleted of Par1b/MARK2 display a significant increase in circularity and phagocytize significantly more neuronal particles than controls. In addition, mice deficient of Par1b show a significant increase in microglia density. Moreover, a hypertrophic phenotype was found in Par1b-deficient mice, with increased branching and shorter processes early in development, and de-ramification with fewer branches and shorter processes in adulthood. This hypertrophic morphology is accompanied by increased phagocytosis of neuronal particles early in development and decreased surveillance of the brain parenchyma. Finally, we show that microglia in Par1b-deficient mice are hypersensitized to injury, indicating that these microglia are in a primed state. Taken together, our results show that Par1 is a key regulator of microglia activation, and depletion of Par1 primes microglia for a hypersensitized response during injury.

## Methods

### Primary microglia cultures

For culturing primary microglia, mixed glia cultures were grown from P1 to P2 Sprague-Dawley rat cortices (Charles River). In short, pups were anesthetized and sacrificed by rapid decapitation. Their brains were extracted and cortices were dissected into Hanks Balanced Salt Solution (HBSS) on ice and cut into small pieces. Cells were dissociated with 1% DNaseI and 2.5% trypsin in HBSS while shaking in a 37 °C water bath. The cell suspension was filtered through a 70 μm nylon cell strainer, counted and plated at about 9 × 10^6^ cells per flask in Minimal Essential Medium Eagle (MEM) (Sigma Aldrich, Saint Louis, MO) supplemented with 10% FBS (Sigma Aldrich, Saint Louis, MO), 3% Glucose, 1 mM Sodium Pyruvate (Invitrogen, Gibco), 1% Glutamax (Invitrogen, Gibco), and 1% Pen/Strep (Invitrogen, Gibco). Media was changed in the flasks at about 5 days post dissection and every 3 days after. Primary microglia were isolated from the mixed glia cultures by shaking at 11–14 days in vitro. They were then seeded on coverslips or culture dishes in astrocyte-conditioned media at the desired density [32].

### Western blotting

Primary microglia cultures were lysed with RIPA buffer (20 mM Tris-HCl, 150 mM NaCl, 0.5% NP40, 1.0% Trition-X 100, 2.0 mM EDTA, 2.0 mM EGTA, and 0.25% DOC) and supplemented with protease inhibitor cocktail (Sigma Aldrich, Saint Louis, MO), phosphatase inhibitor cocktail (Sigma Aldrich, Saint Louis, MO), 10 mM β-glycerophosphate, 10 mM NaF, 10 mM Dithiothreitol (DTT), and 10 mM Phenylmethylsulfonyl fluoride (PMSF). Lysed cells were incubated on ice for 10 min and centrifuged at 13,000×*g* at 4 °C for 10 min. Thirty micrograms cleared lysate was run on a 10% SDS-PAGE gel and transferred to PVDF membrane. Blots were probed with primary antibodies for three out of the four Par1/MARK family members: Par1a/MARK3 (Millipore, Germany: 1:1000; UpState Cell Signaling Solutions, Lake Placid, NY: 1:1000), Par1b/MARK2 (ProteinTech, Rosemont, IL: 1:1000), and Par1c/MARK1 (ProteinTech, Rosemont, IL: 1:1000). Horseradish peroxidase conjugated goat anti-rabbit, and goat anti-mouse antibodies (Jackson ImmunoResearch, West Grove, PA) were used at 1:5000. Imaging of blots by enhanced chemiluminescence was performed using Syngene G:Box/iChemi-XR and the GeneSnap software (Version 7.09.a) (Syngene, Frederick, MD).

### Primary microglia culture transfections and treatment

Polyethylenimine (PEI) was used to transfect primary microglia 3–4 h post-isolation. shRNAs targeting Par1b or luciferase were constructed in the pSUPER-GFP vector and have been described previously [27]. Briefly, PEI (1 mg/ml) was added to serum-free media with desired amount of DNA (1.5:1 PEI:DNA) and allowed to incubate at room temperature for 15 min. The PEI/DNA transfection mixture was then added directly onto cells and allowed to incubate for 2 h. Cells were then washed and incubated in astrocyte-conditioned media for 72 h after transfection. For ATP treatment, microglia were serum starved for 4 h and treated with 50 μM ATP for 5 min. Following incubation, cells were fixed and processed for immunocytochemistry.

### In vitro phagocytosis assay

Primary microglia were transfected as described above. Seventy-two hours post transfection, microglia were serum starved for 4 h. Embryonic day 18 (E18) rat cortices were mechanically dissociated with 2.5% Trypsin and 1% DNase in HBSS. Dissociated cortical neurons were counted and exposed to direct UV light for 30 min, forcibly pipetted and fed onto microglia cultures. Cortical neurons were plated at 4–5 times the density of plated microglia and incubated for 2 h [33]. Following incubation, cells were fixed and processed for immunocytochemistry.

### Immunocytochemistry

Cells on coverslips were fixed with 4% paraformaldehyde/4% sucrose in PBS, permeabilized with 0.2% Triton-X 100 and 5% donkey serum in PBS, and incubated with TuJ1 antibody (BioLegend, San Diego, CA: 1:5000) to label neuronal filaments, or Rhodamine-Phalloidin (Cytoskeleton, Inc., Denver, CO: 1:300) to label the actin cytoskeleton and DAPI to label the nuclei. Cells were washed and incubated with Alexa 594 conjugated donkey anti-rabbit antibody (Jackson ImmunoResearch, West Grove, PA: 1:500; Invitrogen, Carlsbad, CA: 1:500) for 1 h at room temperature. Coverslips were mounted with VectaShield HardSet (Vector Labratories, Burlingmae, CA).

### Animals and genotyping of mouse models

All animal procedures have been approved by the Rutgers-RWJMS Institutional Animal Care and Use Committee. Par1b/MARK2 knockout (KO) (−/−) mice (B6.129X1-*Mark2*^*tm1Hpw*^/J; stock 009365) were crossed with CX3CR1-GFP heterozygous (Het) (+/−) mice (B6.129P-Cx3cr1^tm1Litt^/J; stock 005582) mice. Both lines were purchased from Jackson Laboratories. All animals were housed in a clean, temperature-regulated facility with free access to water and food that maintained a strict 12-h light/dark cycle. Genotyping was performed from genomic DNA extract from mouse-tails and PCR amplified with primer sequences and master PCR protocols supplied from Jackson Laboratories, using the KAPA Mouse HotStart Gentoyping Kit (Kapa Biosystems, Wilmington, MA).

### In vivo two-photon imaging of microglial dynamics

In vivo imaging of microglia dynamics was performed as described previously with modifications [34]*.* Briefly, mice were anesthetized with ketamine (100 mg/kg)/xylazine (10 mg/kg) and administered perisurgical analgesics (0.025% bupivacaine). A small incision was made opening the skin covering each animal’s head to expose the cranium. The cranium was cleaned and dried with 3% hydrogen peroxide. A customized head bar was then glued to the exposed cranium. Excess skin was secured with glue around the headbar to create a waterproof well. A 2.7 mm craniectomy was performed to expose dura mater on the right cortical hemisphere about 2 mm caudal to bregma and 2 mm lateral to the midline by using a hand-held trephine to minimize heat-induced damage to the cortex [35]. Saline was used to keep the exposed brain tissue moist throughout surgery and imaging. This cortical region was selected to be comparable with the traumatic brain injury (TBI) experiments, which are typically performed in this area over the hippocampus, to disrupt congitive function. Thus, we performed all our imaging in this cortical region for consistency.

Following surgery, mice were administered additional ketamine/xylazine and imaged on an Olympus FV1000MPE microscope (Olympus XLPlan N × 25 objective NA 1.05) using a MAITAI DeepSee laser tuned to a wavelength of 900 nm. Time-lapse/Z-stack images were obtained by focusing through the cranial opening on a population of microglia (digitally zoomed to × 50 magnification, 2 μm step size, 10 steps total). To determine microglial dynamics, individual Z-stacks were compressed at each time point and aligned with ImageJ plugin StackReg using the Rigid Body transformation. Each compressed Z-stack was made binary. Binary images were then merged across all time points to reveal the area covered by microglial surveillance during the imaging timeframe. Changes in microglia dynamics was determined by measuring the area covered by microglial surveillance as a percentage of total area.

### Traumatic brain injury (TBI)

Adult mice were subjected to a controlled cortical impact (CCI) injury. CCI was performed as described previously with minor modifications [36]. Briefly, mice were anesthetized with isoflurane and administered perisurgical analgesics (0.025% bupivacaine and buprenorphine (0.1 mg/kg)). A craniectomy was performed to expose the dura mater as described above. The CCI device was placed over the exposed dura mater and lowered until contact was made. A computer-controlled impact was performed with the CCI device. Sample parameters include impact speed 3 m/s, dwell time of 500 ms and cortical deformation of 1 mm. For sham-operated controls, only a craniectomy was performed. Following injury, the skin was re-secured over the skull with Vetbond, and animals were returned to their home cages on heating pads for recovery.

### Perfusion and tissue processing

Animals were anesthetized with ketamine (100 mg/kg)/xylazine (10 mg/kg) and then sacrificed via cardiac perfusion. P5 and P9 animals were perfused by hand with a syringe, and P30 and P120 animals were perfused with a perfusion pump. Once the mice were phosphate-buffered saline (PBS) cleared and fixed with 4% PFA, the brains were extracted and submerged in 4% PFA for 1–2 h post-fixing. Each brain was washed with PBS and transferred through sucrose gradients (10%, 20%, and 30%) for 24 h each at 4 °C. Seventy micrometer, free-floating coronal tissue sections were acquired using a frozen sliding microtome. Sections were stored in cryoprotectant solutions (25% Glycerol, 25% Ethylene Glycol, 30% Sucrose in 0.1 M PBS) until immunohistochemistry was performed.

### Immunohistochemistry (IHC)

For the in vivo engulfment assay, tissue was washed four times with 1× PBS. Following thorough washing, tissue was permeabilized in Blocking Solution (0.3% Triton-X 100, 5% Serum (goat, donkey, or rabbit, etc.), 1× PBS) for at least 1 h at room temperature. Synapsin I and neurofilament-L (NF-L) were immunostained in a stepwise protocol to increase tissue penetration. Briefly, sections were incubated with the synapsin antibody (Millipore, Germany: 1:500) overnight at room temperature (RT). They were then washed and incubated with a DyLight 549 goat anti-rabbit secondary antibody (Jackson ImmunoResearch, West Grove, PA: 1:500; Invitrogen, Carlsbad, CA: 1:500) at RT for 2 h. Following incubation, sections were washed in 1× PBS and re-blocked for at least 1 h at RT. NF-L antibody (Developmental Studies Hybridoma Bank, Iowa City, IA: 1:100) was applied in blocking solution and allowed to incubate overnight at RT. Sections were then washed and incubated with a DyLight 649 horse anti-mouse secondary antibody (Jackson ImmunoResearch, West Grove, PA: 1:500; Invitrogen, Carlsbad, CA: 1:500) at RT for 2 h. After incubation, sections were washed and mounted on charged slides with VectaShield (Vector Laboratories, Burlingame, CA).

For the apoptosis assay, tissue was washed four times with 1× PBS. Following thorough washing, tissue was incubated in Sodium Citrate Buffer at 95 °C for 20 min. Tissue was allowed to cool to room temperature, washed three times with 1× PBS and permeabilized in Blocking Solution (0.2% Triton-X 100, 5% Donkey Serum, in 1× PBS) for at least 1 h at room temperature. Sections were incubated with Cleaved Caspase-3 antibody (Cell Signaling, Danvers, MA; 1:1000) overnight at room temperature (RT). They were then incubated with a Cy3 donkey anti-rabbit secondary antibody (Jackson ImmunoResearch, West Grove, PA: 1:500), washed, and mounted as described above.

For the CCI-TBI experiment, tissue was washed four times with 1× PBS to remove cryoprotectant residue. Following thorough washing, tissue was permeabilized in Blocking Solution (0.3% Triton-X 100, 5% Donkey Serum, 1× PBS) for 30 min at room temperature. Sham-operated control and CCI-TBI tissue was then incubated with donkey-anti-mouse Fab fragment IgG (Jackson ImmunoResearch, West Grove, PA, 1:60 dilution) for at least 1 h at room temperature. Blocking solution was removed from each tissue, and primary antibodies were then applied at the desired dilution to incubate overnight at room temperature. (Iba1, Wako Chemicals USA, Inc., Richmond, VA, 1:1000). The following day, tissue was washed and DyLight 488 Donkey anti-rabbit secondary antibody (Jackson ImmunoResearch, West Grove, PA; 1:500 dilution) and DAPI were applied and incubated at room temperature for 2 h. Tissue was washed and mounted as described above.

### Microscopy and image quantification

To minimize bias in imaging acquisition and quantification, all slides were blinded prior to imaging, and unblinding occurred after image quantification. Fluorescent images were obtained using a Leica DMRE microscope (Leica Microsystems, Inc. Bannockburn, IL) with a 63× oil-immersion lens (Plan Fluorite, NA 1.25) coupled to a Hamamatsu Orca-ER camera (Hamamatsu Photonics, Hamamatsu City, Japan), controlled by OpenLab software (Improvision, Boston, MA). To quantify differences in primary microglia morphology, each individual and isolated microglia cell was traced within ImageJ (version 64bit) and cell circularity (4π × area/perimeter^2^) calculated with the ImageJ program. To quantify the amount of neuronal particles engulfed by microglia, the total number of neuronal particles within each transfected microglia was manually counted.

All tissue sections were imaged on an Olympus FV1000MPE microscope (Olympus UPlan SApo 20x NA 0.75; Olympus LUMPLanFLN × 60 objective NA 1.0). For microglia density analyses, Z-stack images were obtained (digitally zoomed to × 80 magnification, 0.5 μm step size) of the whole 70-μm mounted tissue section sample within the parietal association cortex (bregma − 2 mm; lateral 2 mm), for 2 sections per animal. Each Z-stack was collapsed into a 2D image. Manual counting of individual somas within each image was obtained, including only whole somas within the field.

For microglia morphology analysis in vivo, Z-stack images were obtained by focusing on microglia somas that were in the middle of tissue sample within the parietal association cortex (bregma − 2 mm; lateral 2 mm), to ensure imaging a complete cell (digitally zoomed to × 80 magnification, 0.5 μm step size). All microglia cells that were fully encompassed within the imaged Z-stack were traced and reconstructed using Neurolucida software (Microbrightfield Inc., Colchester, VT) to acquire morphometric analysis including analysis of segmentation, cell bodies, Sholl analyses of branch length, nodes, intersections, and endings.

For analysis of in vivo phagocytosis, 3D surface rendering using the IMARIS software program was performed on individually cropped microglia as described previously [37]. The volume of neuronal material engulfed by microglia was calculated. For cell density quantifications, number of GFP or cleaved caspase-3 positive cells was manually counted within a measured area in ImageJ by an experimenter blinded to the samples.

For analyses of CCI-TBI tissue, × 20 and × 60 images from the cortex Ipsi-laterally to injury were captured. ImageJ was used for density quantifications by manually counting individual Iba1+ cells and measuring total tissue area and the mean fluorescent intensity. For measuring Iba1 fluorescent intensity profile as a function of distance from injury site, a line perpendicular to the brain surface was drawn from the injury site toward the center of the tissue. Fluorescent intensity along the line was plotted as a function of distance away from the surface.

### Cytokine profile array

Brain tissues from postnatal day 5 (P5) Par1b/MARK2 mice were isolated and snap frozen. A mouse cytokine array panel was used to determine cytokine profile (R&D Systems, Minneapolis, MN; Cat# ARY006). Tissue was lysed and processed as per manufacturer’s instructions. Briefly, tissue was homogenized in lysis buffer (PBS with 1% Triton-X 100, protease inhibitor cocktail (Sigma Aldrich, Saint Louis, MO), phosphatase inhibitor cocktail (Sigma Aldrich, Saint Louis, MO), 10 mM β-glycerophosphate, 10 mM NaF, and 10 mM Phenylmethylsulfonyl fluoride (PMSF)) and centrifuged for 10 min at 10,000×g to removed debris. Protein concentration was measured and 500 μg of sample loaded to each blot. Following primary and secondary antibody incubations, blots were exposed for 1, 5, and 10 min. Mean pixel intensities were measured with ImageJ and normalized to positive controls.

### In vivo Evans Blue blood vessel permeability assay

Blood-brain barrier permeability was tested as described previously [38]. Briefly, 200 μl of 0.5% sterile solution of Evans Blue dye dissolved in PBS was injected into the lateral tail vein of Par1b/MARK2 Heterozygous (+/−) and wild-type (+/+) mice. Mice were sacrificed 30 min later by cervical dislocation, and different organs of interest were immediately collected. Organs were placed into 1.5 ml microcentrifuge tubes and weighed. Five hundred microliters of formamide was added to each sample followed by incubation in a 55 °C water bath for 24 h. After incubation, samples were centrifuged, and the supernatant was measured for absorbance at 610 nm. A standard curve was obtained, and the amount of Evans Blue dye extravasated per milligram tissue was calculated.

### Statistical analysis

Statistical analyses were performed using the software StatPlus:mac Pro (version 6.1.60), GraphPad Prism (version 6.0) and/or IBM SPSS (v25). Data are expressed as mean ± standard error mean (SEM). One-way or two-way analysis of variance (ANOVA) was performed, as appropriate, followed by Bonferroni post hoc testing. A *p* value of < 0.05 was considered significant. A linear mixed model in SPSS was utilized when appropriate, and a type III test of fixed effects was used to determine significance.

## Results

### Knockdown of Par1b results in increased activation of primary microglia

To see whether Par1/MARK plays a role in microglia function, we first sought to examine which Par1 family member(s) are expressed in microglia. Previous studies have shown high expression levels of Par1c/MARK1 and Par1b/MARK2 in the brain. Par1a/MARK3 also shows significant expression in the brain while Par1d/MARK4 expression is virtually non-detectable in the normal brain [39, 40]. However, whether any of these family members are expressed in the microglia remains to be determined. We thus examined the expression of Par1 family members in primary hippocampal neurons, astrocytes, and microglia cultures, following confirmation of culture purity (Additional file 1: Figure S1). Both Par1c/MARK1 and Par1a/MARK3 are expressed mainly in neurons and/or astrocytes with little expression in microglia. However, Par1b/MARK2 shows relatively uniform expression in all cell types (Additional file 1: Figure S2). These results are consistent with previously reported RNAseq transcriptome analysis of different cell types in the cerebral cortex [41]. Thus, we have focused our studies on the Par1b/MARK2 family member.

**Fig. 1:**
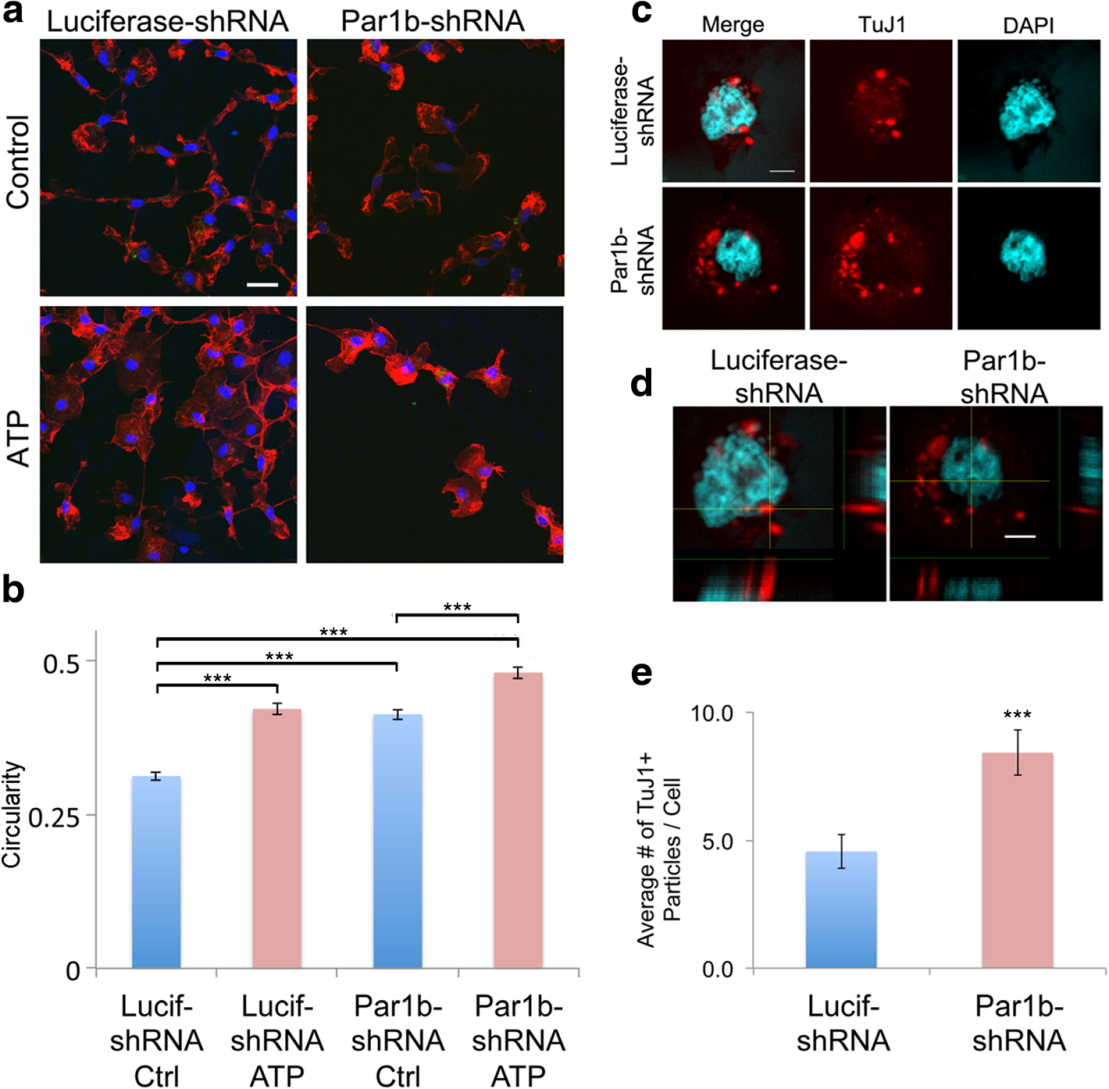
Knockdown of Par1b significantly activates primary microglia. **a** Primary microglia were transfected with shRNA targeting either luciferase or Par1b shRNA. Seventy-two hours post-transfection, cells were serum starved, stimulated with 50 μM ATP, fixed, and immunostained with Rhodamine-Phalloidin (red) and DAPI (blue). Scale bar = 50 μm. **b** Quantification of microglial circularity (4π × area/perimeter^2^) under conditions described in a. *n* = 472 cells for Lucif-shRNA Ctrl, 542 cells for Lucif-shRNA ATP, 522 cells for Par1b-shRNA Ctrl, and 483 cells for Par1b-shRNA ATP, from three independent experiments, ***p < 0.001 by two-way ANOVA. **c** Primary microglia were transfected with different shRNAs as in a. Seventy-two hours post-transfection, cells were serum starved, incubated for 2 h with UV damaged neurons, fixed, and immunostained with TuJ1 (red) and DAPI (blue). Scale bar = 5 μm. **d** Orthogonal view shows that the TuJ1+ particles are within the microglia. Scale bar = 5 μm. **e** Quantification of the number of TuJ1+ particles per cell. *n* = 37 cells for Lucif-shRNA and 34 cells for Par1b-shRNA from two independent experiments, ***p < 0.001 by one-way ANOVA

To see if Par1b/Mark2 plays a role in microglia regulation, a short hairpin RNA (shRNA) was transfected into primary microglia cultures to knockdown expression of endogenous Par1b/MARK2 [27]. An shRNA targeting luciferase was used as a control. Cells were incubated for 72 h after transfection. The efficiency of transfection was found to be greater than 90%. The cells were then fixed and stained with Rhodamine-Phalloidin (red) to label the actin cytoskeleton and DAPI (blue) to label the nuclei (Fig. 1a). In control microglia expressing an shRNA targeting luciferase, the cells show an elongated or triangular shape typical of these cells in culture. Upon stimulation with ATP, a known activator of microglia, cells show a dramatic morphological change with a more rounded shape, and ruffling of the cell periphery. Quantifications were performed to measure cell circularity, which has a range from 0 to 1, with 1 representing a perfect circle. As expected, ATP treatment caused a significant increase in circularity in luciferase shRNA transfected microglia (Fig. 1b and Additional file 1: Table S1a, ***p < 0.001). Strikingly, microglia depleted of Par1b show a rounded morphology and membrane ruffling even in the absence of ATP stimulation, with further rounding observed upon ATP treatment (Fig. 1b and Additional file 1: Table S1a, ***p < 0.001). These results suggest microglia depleted of Par1 display a rounded morphology reminiscent of activated microglia.

We next sought to evaluate if and how Par1 affects functional microglia activation. Phagocytosis is a key function of microglia, allowing them to clear cellular debris from the CNS. Following injury or illness, damaged neuronal particles are a natural target of microglia. Thus, we assessed the ability of Par1b-depleted primary microglia to phagocytize UV damaged neuronal particles. Primary microglia were again transfected with shRNAs targeting luciferase or Par1b. After 72 h, microglia were incubated with UV damaged neurons, fixed and probed for neuronal marker TuJ1 (red), and the nuclear marker DAPI (blue) (Fig. 1c). Confocal Z-stacks were taken to ensure the neuronal particles reside within the microglia (Fig. 1d). Microglia depleted of Par1 phagocytized almost twice as many TuJ1-positive neuronal particles compared to controls (Fig. 1e and Additional file 1: Table S1b, ***p < 0.001). This suggests decreased expression of Par1 results in increased phagocytosis of neuronal particles by microglia.

### Loss of Par1b/MARK2 increases microglia density and alters microglia morphology during brain development in vivo

Our in vitro results suggest Par1b/MARK2 regulates microglia activation. Depletion of Par1b resulted in a rounded morphology of microglia and increased phagocytosis. We wanted to confirm these results and delve further into the in vivo regulation of microglia by Par1b. First, we crossed Par1b/MARK2 knockout mice with CX3CR1-GFP mice, in which all microglia and other immune cells express GFP [42]. It has previously been shown that microglia undergo significant changes in their density, morphology, and phagocytic abilities throughout development, which is important for their role in sculpting the developing brain [9, 10]. Thus, we examined microglia in the CX3CR1-GFP-positive Par1b/MARK2 KO mice across different developmental time points.

**Fig. 2:**
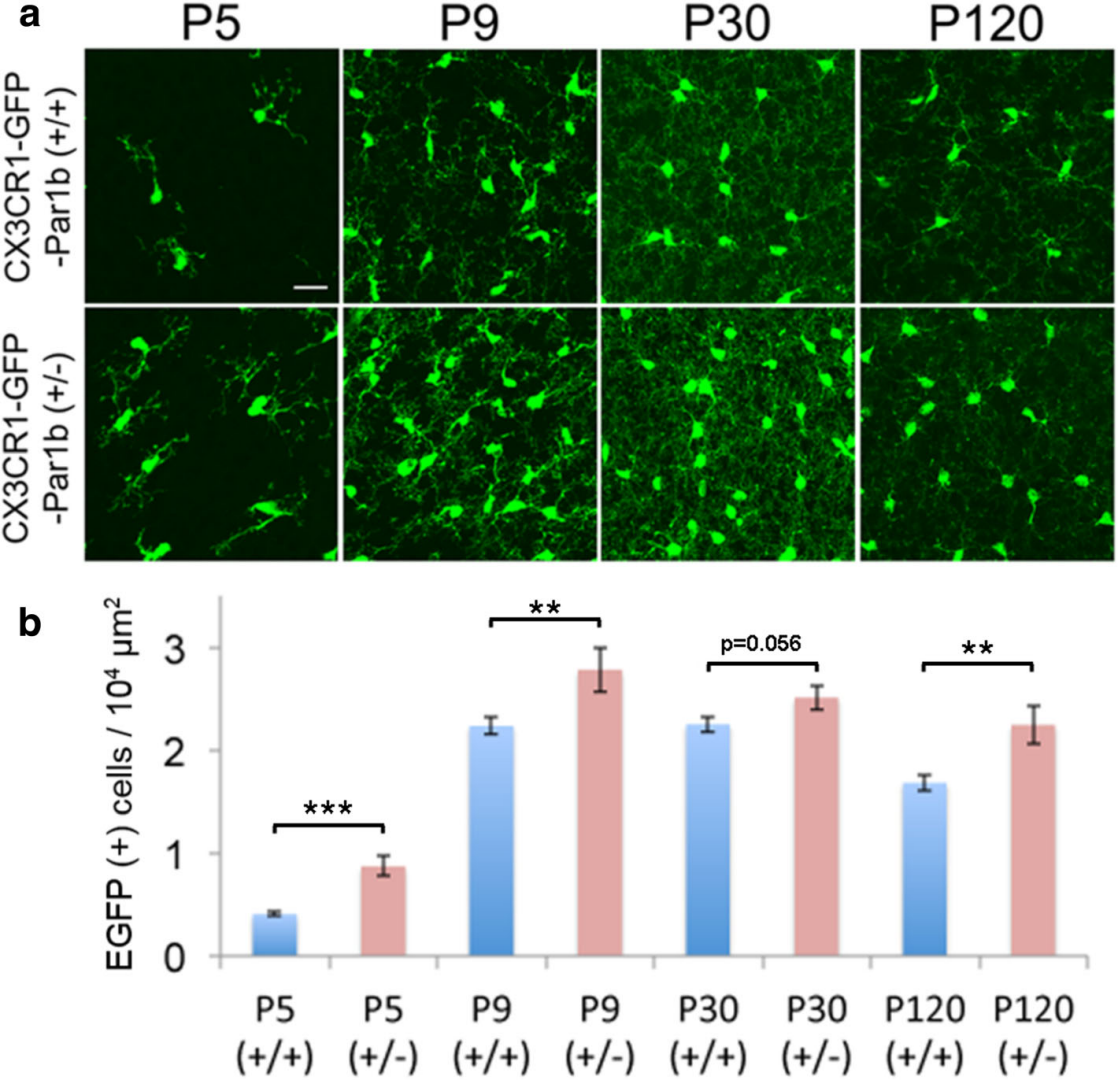
Loss of Par1b increases microglia density in vivo. **a** Representative images of CX3CR1-GFP-positive Par1b (+/+) and (+/−) mice at P5, P9, P30, and P120 within the parietal association cortex. Scale bar = 20 μm. **b** Quantification of microglial density. CX3CR1-GFP-positive Par1b (+/−) mice show a significant increase in microglia density at P5, P9, and P120, with the trend holding at P30. *n* (animals) = Par1b (+/+) P5(6), P9(7), P30(5), P120(7); Par1b (+/−) P5(7), P9(3), P30(5), P120(5), **p < 0.01, ***p < 0.001 by one-way ANOVA

We first sought to examine if mice deficient of Par1b/MARK2 have alterations in the density of microglia within the cortex. Microglia density is tightly regulated throughout development and having an appropriate amount of microglia is important for maintaining brain homeostasis throughout life [43–45]. To determine the effects of Par1b on microglia density, Par1b (+/+) mice were compared to Par1b (+/−) mice at the various developmental time points (Fig. 2a) for changes in microglia density within the parietal association cortex. Interestingly, a significant increase of microglia density was observed in Par1b (+/−) mice compared with Par1b (+/+) mice at P5, P9, and P120, with the trend holding at P30 (Fig. 2b and Additional file 1: Table S2, **p < 0.01, ***p < 0.001).

**Fig. 3:**
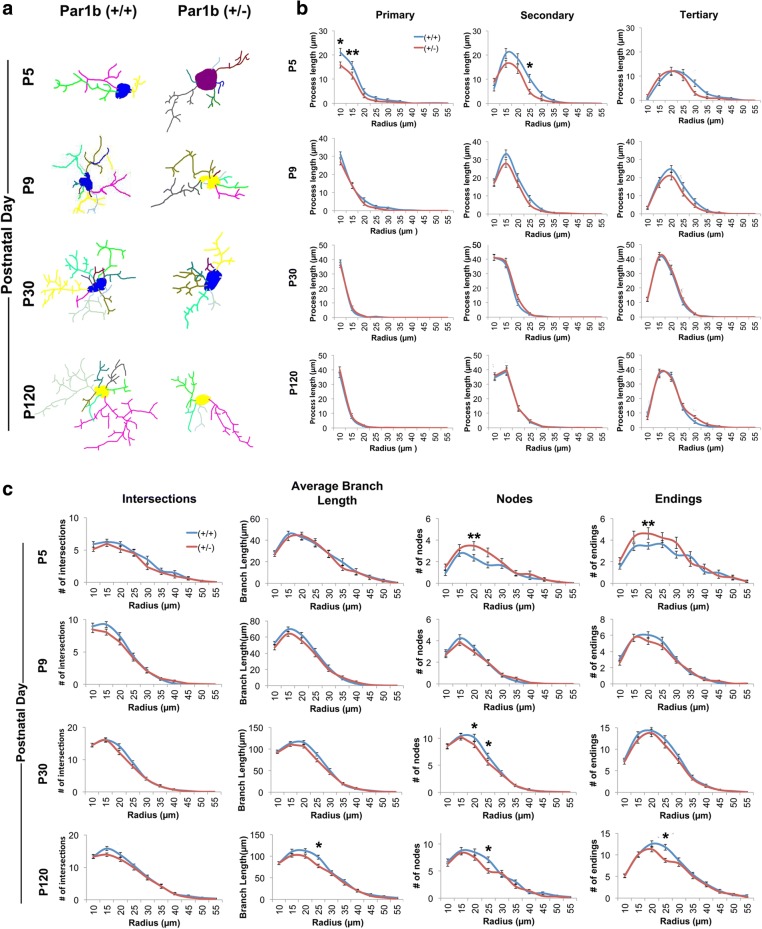
Loss of Par1b/MARK2 alters microglia morphology during brain development in vivo. **a** Representative 3D reconstructions of microglia from CX3CR1-EGFP-positive Par1b (+/+) and (+/−) mice at P5, P9, P30, and P120 within the parietal association cortex. **b** Branch complexity was quantified utilizing a Sholl analysis with a somal radius of 10 μm, measuring total lengths of primary, secondary, and tertiary processes. **c** Morphological aspects were quantified utilizing a Sholl analysis with a somal radius of 10 μm, measuring for number of intersections, average branch length, nodes, and endings. *n* (animals/microglia) = Par1b (+/+): P5(4/40), P9(4/43), P30(5/41), P120(5/37); Par1b (+/−): P5(4/36), P9(3/39), P30(4/39), P120(3/37), *p < 0.05, **p < 0.01 by type III test of fixed effects using linear mixed model analyses

We next asked if these microglia displayed a different morphological profile at the various developmental time points. To assess this, Z-Stack images were obtained from the parietal association cortex of each animal, 3D reconstructions were created using the Neurolucida program, and Sholl analysis was performed on the reconstructed microglia. Various morphological aspects were measured, including number of nodes, intersections, endings, average branch length, and total lengths of primary, secondary, tertiary (Fig. 3 and Additional file 1: Table S3a and S3b) and quaternary processes (Additional file 1: Figure S3, Table S3a and S3b). Interestingly, we found that microglia with reduced Par1b expression display increased nodes and endings at P5, but reduced primary and secondary process lengths early in development. In adult Par1b (+/−) mice, microglia show a de-ramified phenotype with shorter processes and fewer nodes and endings. Together, these results indicate that microglia with reduced Par1b show a hypertrophic phenotype with increased branching and shorter processes early in development, while they become de-ramified in adulthood with fewer branches and shorter processes. These morphological changes are reminiscent of microglia in their “primed” state, which shows a lower threshold of activation.

### Loss of Par1b/MARK2 increases microglia phagocytosis during postnatal development

**Fig. 4:**
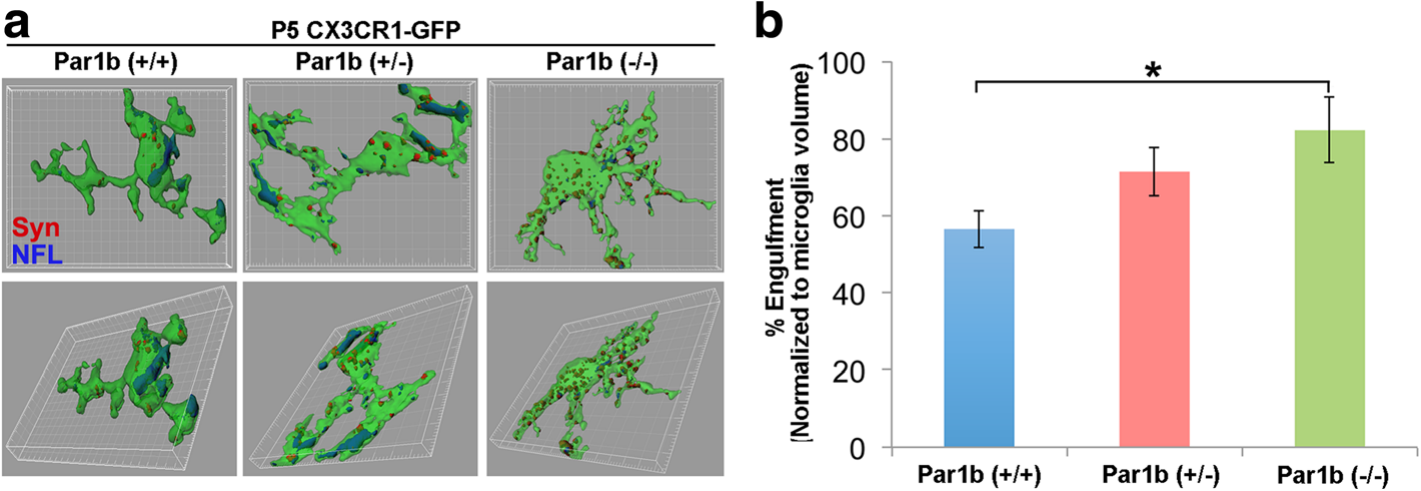
Loss of Par1b/MARK2 increases microglia phagocytosis during postnatal development. **a** Representative 3D surface renderings of individual microglia (green) with engulfed neuronal particles (neurofilament, blue; synapsin1, red) from the parietal association cortex. **b** Quantifications of neuronal material phagocytized within each microglia. Par1b (−/−) microglia were found to engulf more neuronal particles than Par1b (+/+), *n* (animals/microglia) = Par1b (−/−): (2/34); Par1b (+/−): (7/44); Par1b (+/+) (6/39), *p < 0.05 by one-way ANOVA

Our above in vitro findings indicate microglia depleted of Par1 phagocytize significantly more neuronal particles than controls (Fig. 1). Since microglia in the Par1b (+/−) mice show an altered morphological profile during development, which indicates activation of these cells, we next asked whether these microglia show changes in their ability to phagocytize neuronal particles. During brain development, neurons undergo significant programmed cell death from embryonic stages through the first few postnatal weeks. Moreover, microglia actively prune synapses through phagocytosis during the first postnatal week [10]. Thus, we examined microglia phagocytosis of different neuronal particles within the parietal association cortex at P5, a critical time-point known for neuronal and synaptic pruning. CX3CR1-GFP-positive Par1b (+/+), (+/−), and (−/−) mice were collected at P5 co-immunostained with the synaptic marker synapsin I, and the neuronal intermediate filament marker, neurofilament-L. 3D reconstruction confirmed that P5 microglia engulf numerous synaptic and other neuronal particles (Fig. 4a). The amount of engulfed neuronal materials significantly increased in the Par1b (−/−) mice as compared with the Par1b (+/+) mice (Fig. 4b and Additional file 1: Table S4, *p < 0.05), which indicates that microglia without Par1b show increased phagocytic abilities.

### Loss of Par1b/MARK2 reduces microglia dynamics in vivo

**Fig. 5:**
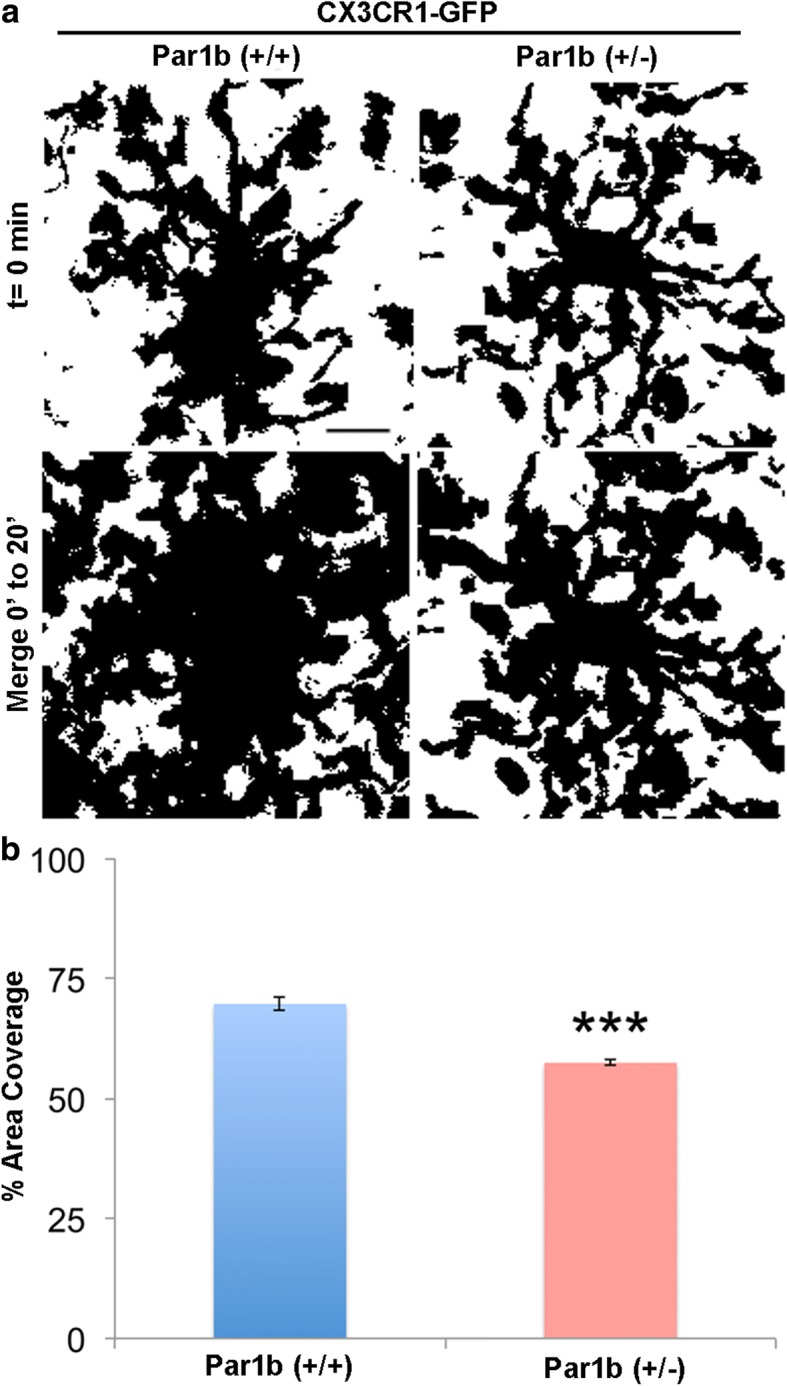
Loss of Par1b/MARK2 reduces microglia dynamics in vivo. **a** Time-lapse Z-stack images were acquired through an open cranial window at the parietal association cortex in anesthetized CX3CR1-GFP-positive Par1b (+/+) and (+/−) mice. Each Z-stack was compressed and made binary. Binary images from each time point (10 total over 20 min) were merged into one final image (merge 0′–20′) to reveal area surveyed by microglia during the imaging timeframe. Scale bar = 10 μm. **b** The percent area coverage of an individual microglia’s processes was measured. A significant decrease in the percent area surveyed by Par1b (+/−) mice was found when compared to Par1b (+/+) mice, indicating decreased process dynamics. *n* (animals/microglia) = Par1b (+/+): (6/60); Par1b (+/−): (7/70), ***p < 0.001 by one-way ANOVA

Microglia processes have been found to be highly motile and dynamic, allowing for their constant surveillance of the CNS. Recent studies show decreased ramification results in a reduction of surveillance by microglia processes [46]. Our current findings show Par1b (+/−) mice have an increase in microglia density, alterations in microglia morphology, and increased neuronal engulfment, all reminiscent of microglia in their “primed” state. To see if these microglia show any changes in surveillance under physiological conditions, we live imaged the dynamics of microglia processes in adult CX3CR1-GFP-positive Par1b (+/+) and (+/−) mice through an open cranial window. To quantify microglia dynamics, time-lapse Z-stacks were acquired. Images were made binary and merged across all time points to show the area scanned by microglia over the imaging time. Interestingly, Par1b (+/−) mice were found to have a significant decrease in percent area scanned when compared to Par1b (+/+) mice, showing that these cells exhibit lower dynamics of their processes and lower surveillance activity (Fig. 5 and Additional file 1: Table S5, ***p < 0.001).

### Loss of Par1b increases microglia activation in response to controlled cortical impact injury in mice

**Fig. 6:**
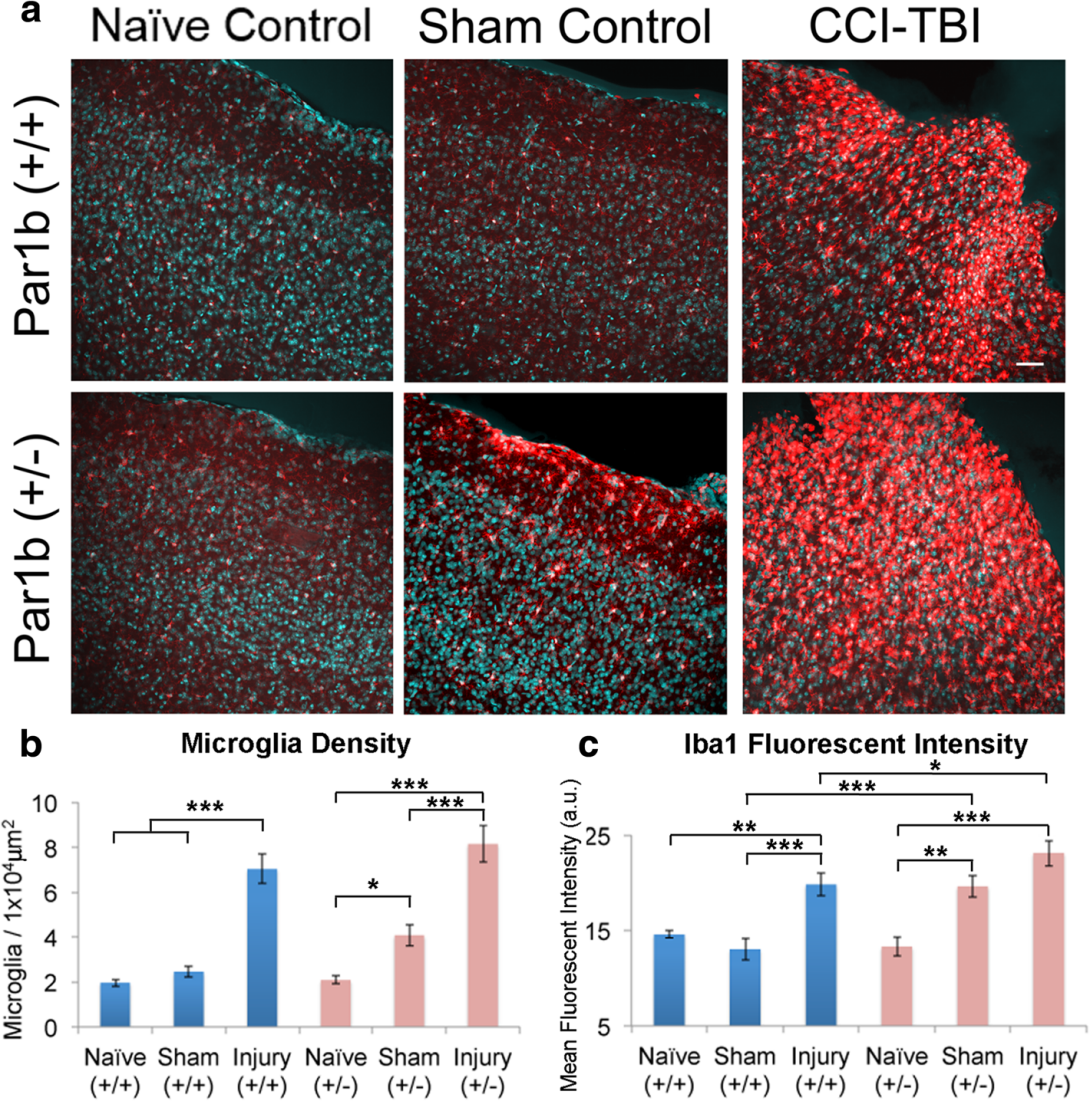
Loss of Par1b increases microglia activation in response to controlled cortical impact injury in mice. **a** Representative 20× confocal images of the parietal association cortex around the impact region in CCI, sham-operated, and naïve control Par1b WT (+/+) and Het (+/−) mice 7 days post-surgery. The brains were sectioned and immunostained for microglia marker Iba1 (red) and DAPI (blue). Scale bar = 50 μm. **b** Quantification of microglial density, *n* = Par1b (+/+): naïve (5), sham (3), TBI (6); Par1b (+/−):naïve (3), sham (3), TBI (6), *p < 0.05, ***p < 0.001 by two-way ANOVA. **c** Quantification of fluorescent intensity of Iba1+ cells, *n* (animals) = Par1b (+/+): naïve (5), sham (3), TBI (6); Par1b (+/−): naïve (3), sham (3), TBI (6)), *p < 0.05, **p < 0.01, ***p < 0.001 by two-way ANOVA

The above data indicate that microglia in the Par1b (+/−) mice show a phenotype consistent with primed microglia in the aging or immune-challenged brain. Since microglia priming usually results in a lower threshold for activation upon insult, we examined microglia response in a controlled cortical impact (CCI) model of traumatic brain injury (TBI). As expected following a TBI, both Par1b (+/+) and (+/−) mice show a robust microglia response at the injury site, with both having a comparable increase in microglia density, as shown by immunostaining with the microglia marker Iba1 (Fig. 6a, b and Additional file 1: Table S6a). These densely populated microglia exhibit a rounded, amoeboid morphology at the injury site. To verify this activated microglial response, we also measured for alterations in overall Iba1 fluorescent intensity. Interestingly, we found Par1b (+/−) injured mice exhibited a significant increase in overall fluorescent intensity of microglia when compared to Par1b (+/+) injured mice (Fig. 6a, c and Additional file 1: Table S6b, *p < 0.05). Moreover, this increased Iba1 fluorescent intensity in Par1b (+/−) injured mice was found to extend significantly further away from the injury surface than Par1b (+/+) injured mice (Additional file 1: Figure S4, *p < 0.05), indicating that microglia located more distal from the injury site are significantly more activated in the Par1b (+/−) injured mice.

Remarkably, a substantial amount of microglia activation was found in the sham-operated Par1b (+/−) mice, with microglia displaying a rounded morphology at the peripheral site where the craniectomy was performed. We found almost a 2-fold increase in microglia density in the Par1b (+/−) sham-operated mice compared to the Par1b (+/+) sham-operated control and the Par1b (+/−) naïve control. However, no noticeable microglia activation was observed in the sham-operated Par1b (+/+) mice (Fig. 6b and Additional file 1: Table S6a, **p < 0.01, ***p < 0.001). These results suggest that microglia are hypersensitized when Par1b expression is reduced. To verify this hypersensitized microglia response, we also measured for alterations in overall fluorescent intensity of the microglia. The changes found in fluorescent intensity mirrors the density alterations, with Par1b (+/−) sham-operated control animals yielding a significant increase compared to Par1b (+/+) sham-operated controls (Fig. 6c and Additional file 1: Table S6b, **p < 0.01, ***p < 0.001). Taken together, these results indicate that microglia activation is facilitated by the loss of Par1b.

### Altered microglia morphology and function in the Par1b (+/−) mice are likely due to a cell-autonomous mechanism within the microglia

Since the Par1b/MARK2 KO mice are a constitutive knockout model, we wanted to confirm that the alterations in microglia morphology and function are due to intrinsic mechanisms within the microglia and are not indirect effects of increased neuronal death or changes in the cytokine milieu. To examine this, we first immunostained for cleaved caspase-3 in the P5 brain. As seen in Additional file 1: Figure S5, Par1b (+/−) brain shows comparable apoptosis in different brain regions as the (+/+) counterpart, which shows that the observed microglia activation is not due to increased neuronal death (Additional file 1: Table S7a).

Next, we wanted to see whether there are changes in the cytokine milieu in the brain. Cytokines in the brain parenchyma can be secreted by astrocytes, microglia, or neurons. Changes in the cytokine profile secreted by other cell types can potentially activate microglia independent of cell-autonomous changes within microglia. Thus, we examined the cytokine profile of Par1b WT and KO brains at P5. No significant differences in the cytokine profile was observed (Additional file 1: Figure S6), suggesting that microglia activation observed in the KO brain was not due to an indirect effect of altered cytokine secretion.

Finally, we wanted to confirm that microglia activation was not due to the infiltration of peripheral immune cells or inflammatory mediators as a result of compromised blood-brain barrier (BBB). To determine if Par1b-deficient mice have an intact BBB, we used an in vivo Evans Blue blood vessel permeability assay comparing the Par1b (+/+) versus (+/−) mice. No significant change in the concentration of Evans Blue dye were observed in the liver, lung, small intestine, skin, kidney and brain (Additional file 1: Figure S7 and Table S7b). This suggests the BBB of Par1b (+/−) mice is intact.

## Discussion

Microglia are increasingly recognized as an integral partner of the CNS for early development and throughout life. Yet, the mechanisms regulating microglia activation remain poorly understood. Here, we show that Par1b/MARK2 regulates microglia activation both in vitro in primary microglia cultures, and in vivo in a Par1b/MARK2 KO mouse model. We found that shRNA knockdown of Par1b in primary microglia results in a rounded morphology and increased phagocytosis. In P5 Par1b/MARK2 knockout mice, microglia exhibit shortened primary and secondary processes but increased branching, which is reminiscent of hypertrophic microglia observed in autism spectrum disorders (ASD) [47], aging [48, 49], or epilepsy [50].

Microglia that display a hypertrophic morphology are believed to be primed and hyper-responsive to insults. Primed microglia have a lower threshold to switch to the fully activated, amoeboid state. Consistent with this idea, microglia with reduced Par1b display heightened sensitivity to injury, and injured Par1b (+/−) mice show increased microglia activation distal to the injury site. Remarkably, sham operated Par1b (+/−) mice show significant levels of microglia activation. This suggests that microglia with reduced Par1b expression are primed and hypersensitive to insults similar to microglia in the aged brain. It would be of great interest to examine whether there are changes in Par1 activity/expression during aging, and whether these changes are responsible for the age-related changes in microglial activation.

Under physiological conditions, microglia constantly extend and retract their processes to scan the brain environment [51]. Microglial processes make regular contacts with neuronal synapses, which is important for neuronal homeostasis and synaptic plasticity [11, 15, 52]. However, how microglial dynamics is regulated remain unclear. Interestingly, we found that microglia in Par1b (+/−) mice show decreased dynamics under homeostatic conditions. This decrease in dynamics coupled with shortened processes results in reduced surveillance of the brain parenchyma, which could have consequences for neuronal homeostasis and plasticity. Indeed, previous studies show that microglia process motility decreases in the aging brain [53–55] and is impaired in certain pathological conditions including Alzheimer’s disease [56, 57] and Huntington’s disease [58]. Together, our results indicate that microglia in mice with reduced Par1b expression show a phenotype consistent with “primed” microglia in the aging brain, with decreased surveillance activity and a lower threshold for activation upon injury.

What might be the underlying molecular mechanisms mediating the role of Par1 in microglia function? Our data indicate that reduced Par1b expression increased microglial density throughout development. This is likely a result of increased microglial proliferation. One of the downstream effectors of Par1b/MARK2 is Cdc25, a cell cycle regulator that functions to promote cell cycle progression. Par1b phosphorylates Cdc25, which creates a binding site for 14–3-3 and inhibits Cdc25 activity by sequestering it in the cytoplasm [59]. It is possible that this inhibitory mechanism is impaired in microglia with reduced Par1b expression, leading to increased proliferation and microglial density.

Our data show that both primary microglia and microglia in vivo display activated morphology and increased phagocytosis of neuronal particles. This would likely involve changes in cellular processes such as protrusion extension/retraction, branching, and phagocytic cup formation. Each of these processes would require intricate collaborations between the actin and microtubule cytoskeletal networks. Interestingly, many of the substrates of Par1/MARK are regulators of the actin or microtubule cytoskeleton, such as microtubule-associated proteins MAP2 and tau [40], doublecortin [23, 60], and plakophilin [61]. It is possible that depletion of Par1b affects these microglial processes through modulating actin and microtubule dynamics.

## Conclusions

In summary, our data show a novel role for Par1b/MARK2 in regulating microglia function during development and under injury conditions. Microglia with reduced Par1b expression show changes in morphology consistent with a hypertrophic phenotype, increased phagocytosis of neuronal particles, decreased surveillance of the brain parenchyma, and lower threshold of activation upon injury. These phenotypes are consistent with primed microglia in a number of neurological disorders as well as the aging brain. It would be of great interest to decipher the role of Par1 in microglial activation under these physiological and pathological conditions.

## Additional file

Additional file 1: **Figure S1.** Purity of primary microglia cultures. **Figure S2.** Par1b/MARK2 is expressed in microglia. **Figure S3.** Microglia morphology in Par1b deficient mice. **Figure S4.** Microglia located more distal from the injury site are significantly more activated in the Par1b (+/−) mice. **Figure S5.** No significant increase in apoptosis was observed in Par1b KO brains. **Figure S6.** No significant change in cytokine profiles in P5 Par1b (+/+) versus Par1b (−/−) brains. **Figure S7.** No significant change in extravasation of Evans Blue dye was found in different organs from Par1b (+/+) versus (+/−) mice. **Table S1a.** Primary microglia circularity. **Table S1b.** Primary microglia engulfment of neuronal particles. **Table S2.** Microglia density during development. **Table S3a.** Microglia developmental morphology—branch complexity. **Table S3b.** Microglia developmental morphology—structural characteristics. **Table S4.** Early microglia engulfment of neuronal particles. **Table S5.** Microglia dynamics. **Table S6a.** Microglia density post-TBI. **Table S6b.** Overall microglia fluorescent intensity post-TBI. **Table S7a.** Apoptosis assay. **Table S7b.** Blood vessel permeability assay. (PDF 1967 kb)

## Abbreviations

ATP: Adenosine triphosphate
BBB: Blood-brain barrier
CCI: Controlled cortical impact
Cdc25: Cell division cycle 25
CNS: Central nervous system
CX3CR1: C-X3-C motif chemokine receptor 1
GFP: Green fluorescent protein
KO: Knockout
MARK2: Microtubule affinity regulating kinase 2
Par1b: Partitioning defective 1b
PFA: Paraformaldehyde
shRNA: Short hairpin RNA
TBI: Traumatic brain injury
