# Supplementary material for: Loss of Par1b/MARK2 primes microglia during brain development and enhances their sensitivity to injury
Source: J Neuroinflammation. 2019 Jan 17;16:11. doi: 10.1186/s12974-018-1390-3 (PMC6335724; doi:10.1186/s12974-018-1390-3)
Supplement: Supplementary file 1 — Figure S1. Purity of primary microglia cultures. Figure S2. Par1b/MARK2 is expressed in microglia. Figure S3. Microglia morphology in Par1b deficient mice. Figure S4. Microglia located more distal from the injury site are significantly more activated in the Par1b (+/−) mice. Figure S5. No significant increase in apoptosis was observed in Par1b KO brains. Figure S6. No significant change in cytokine profiles in P5 Par1b (+/+) versus Par1b (−/−) brains. Figure S7. No significant change in extravasation of Evans Blue dye was found in different organs from Par1b (+/+) versus (+/−) mice. Table S1a. Primary microglia circularity. Table S1b. Primary microglia engulfment of neuronal particles. Table S2. Microglia density during development. Table S3a. Microglia developmental morphology—branch complexity. Table S3b. Microglia developmental morphology—structural characteristics. Table S4. Early microglia engulfment of neuronal particles. Table S5. Microglia dynamics. Table S6a. Microglia density post-TBI. Table S6b. Overall microglia fluorescent intensity post-TBI. Table S7a. Apoptosis assay. Table S7b. Blood vessel permeability assay. (PDF 1967 kb) [file 12974_2018_1390_MOESM1_ESM.pdf]

Supplementary Information

Loss of Par1b/MARK2 primes microglia during brain development and enhances their sensitivity to injury

Victoria L. DiBona, Wenxin Zhu, Mihir K. Shah, Aditi Rafalia, Hajer Ben Cheikh, David P. Crockett, and Huaye Zhang*

Department of Neuroscience and Cell Biology, Robert Wood Johnson Medical School, Rutgers, The State University of New Jersey, Piscataway, NJ, USA

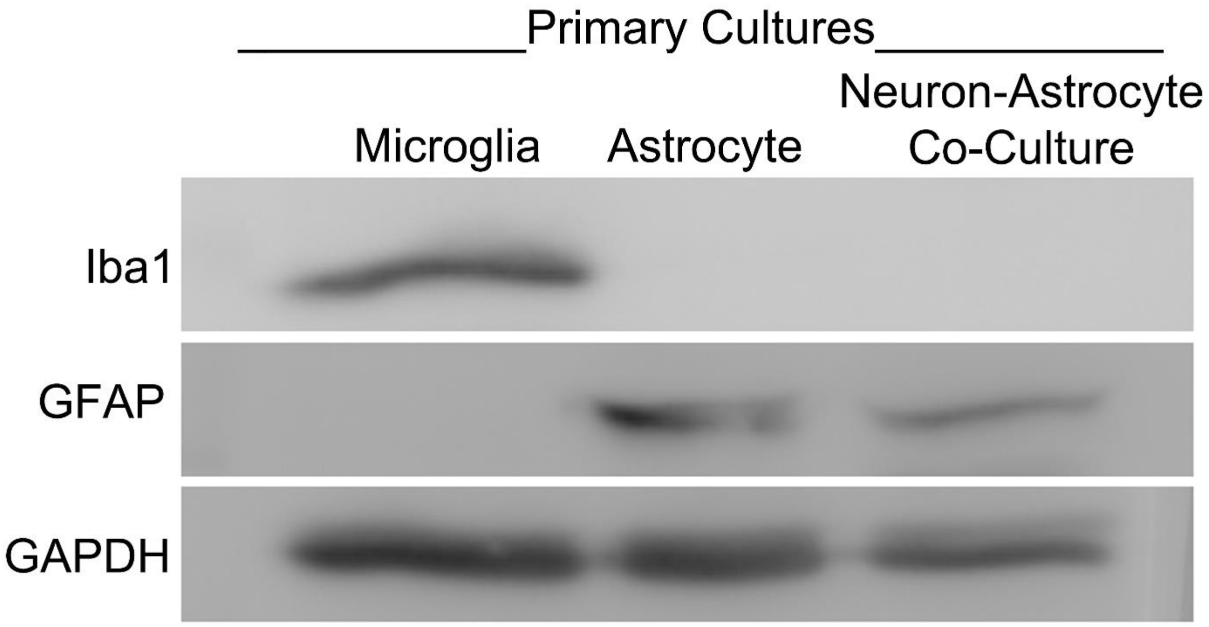

Figure S1. Purity of primary microglia cultures. Protein lysates of primary microglia, astrocytes, and cortical neuron-astrocyte co-cultures were analyzed by Western blot for microglia marker Iba1, astrocyte marker GFAP, and GAPDH as a loading control. Microglia cultures were found to be pure from astrocyte contamination.

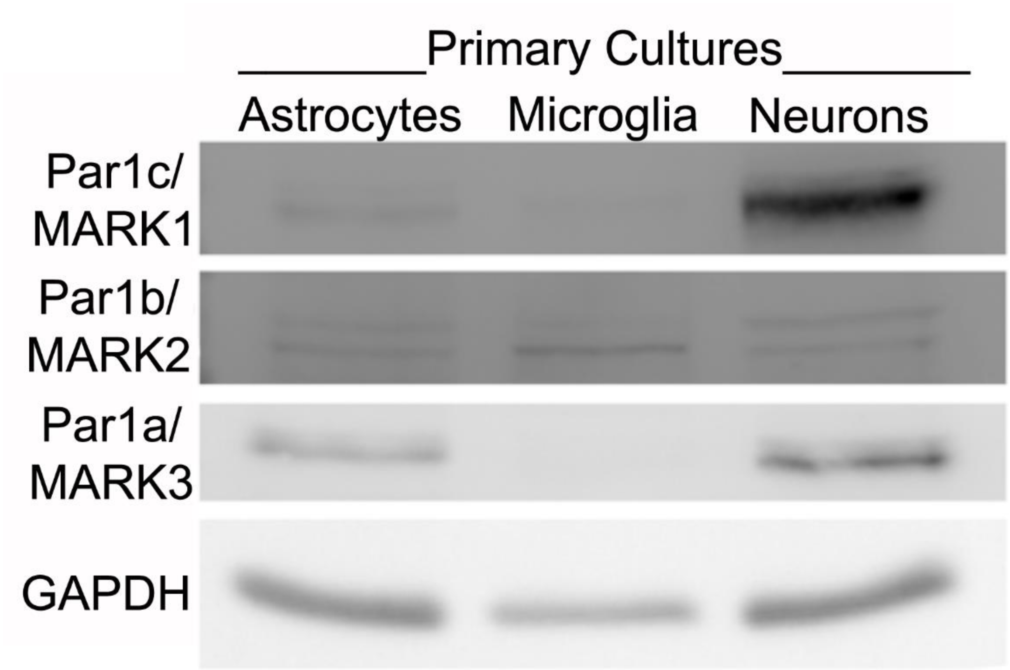

Figure S2. Par1b/MARK2 is expressed in microglia. Protein lysates of primary microglia, primary neurons and astrocytes cultures were analyzed by Western blot for Par1/MARK family members and loading control GAPDH.

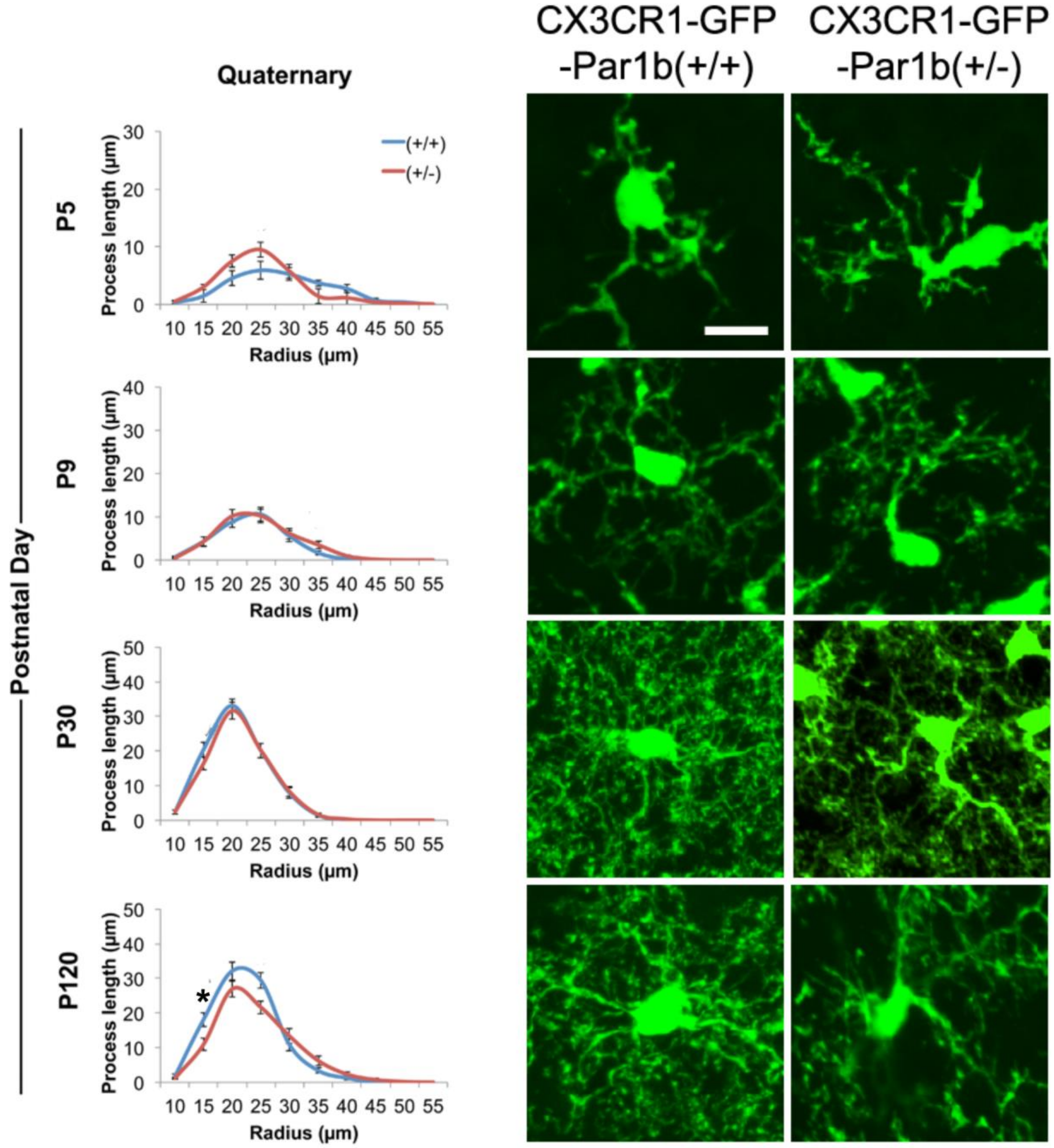

Figure S3. Microglia morphology in Par1b deficient mice. Left panel: Quantifications of quaternary processes in microglia from CX3CR1-EGFP-positive Par1b (+/+) and (+/-) mice at P5, P9, P30 and P120. Right panel: Representative images of microglia in Par1b (+/+) and (+/-) mice at different developmental time points. Scale bar: 20 µm.

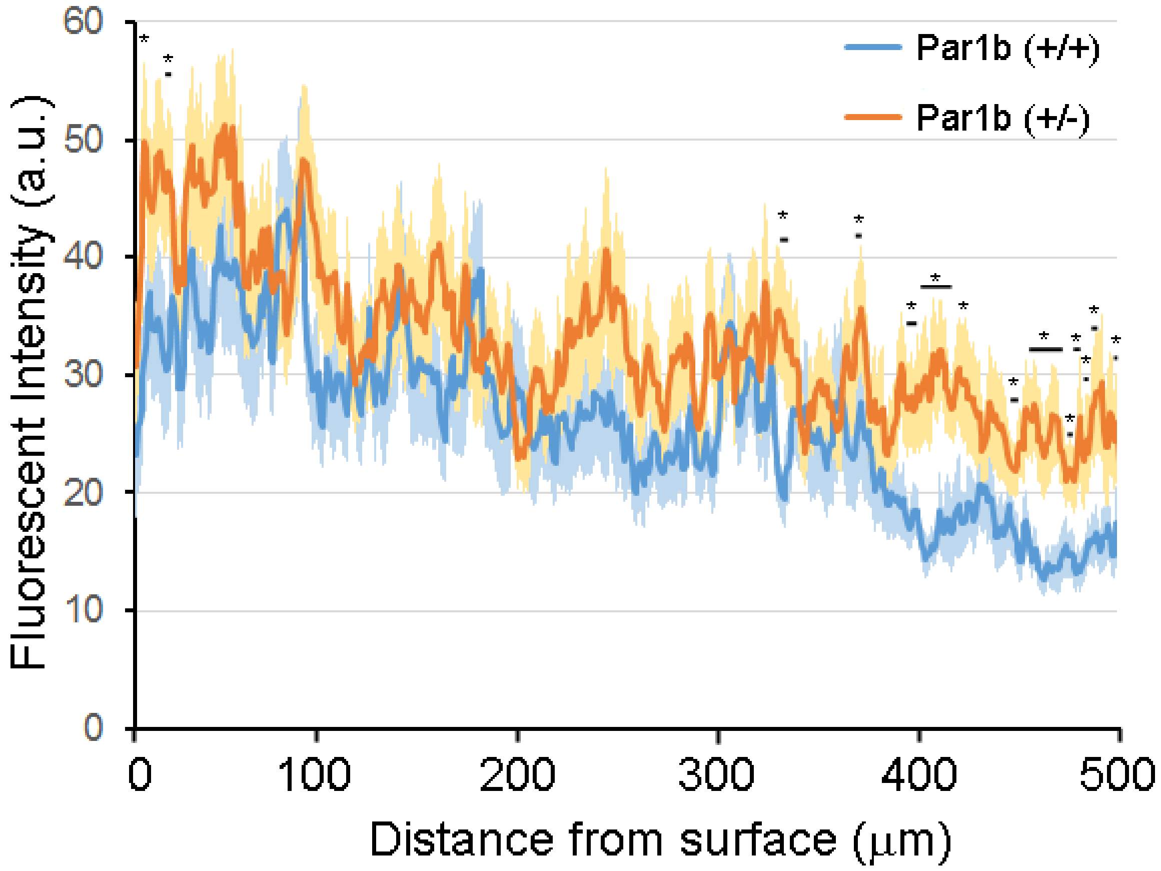

Figure S4. Microglia located more distal from the injury site are significantly more activated in the Par1b (+/-) mice. Line intensity profile of Iba1 fluorescence was measured by drawing a line perpendicular to the brain surface from the injury site toward the center of the tissue. Fluorescent intensity of Iba1 along the line was measured by ImageJ. *$p<0.05$.

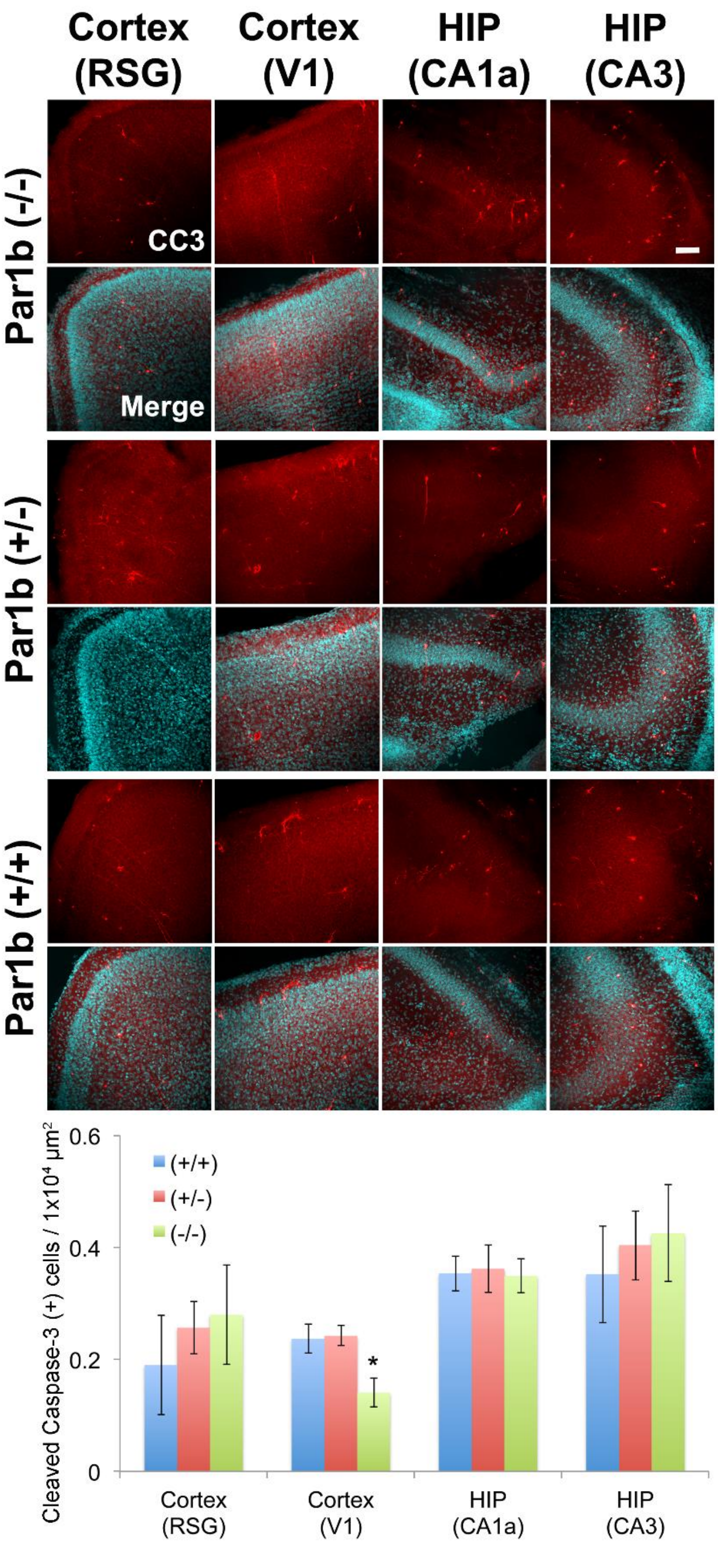

Figure S5. No significant increase in apoptosis was observed in Par1b KO brains. Par1b (+/+), (+/-) and (-/-) tissue was immunostained with cleaved caspase-3 (red) and DAPI (cyan). Lower panel shows quantification of apoptotic cells in different brain regions, n (animals) = Par1b (-/-): 3; Par1b (+/-): 5; Par1b (+/+): 4, *$p<0.05$. Scale bar: 50 µm.

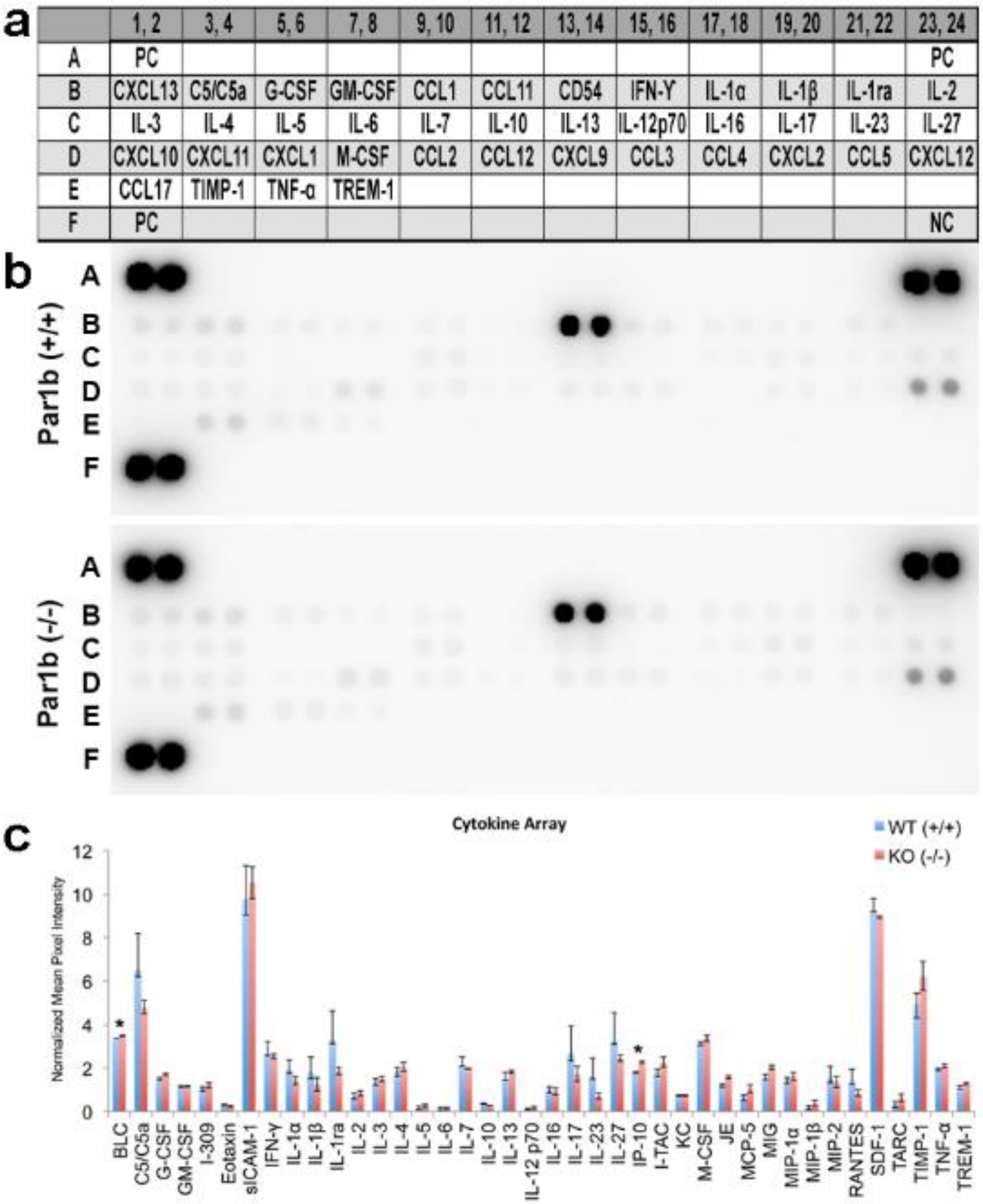

Figure S6. No significant change in cytokine profiles in P5 Par1b (+/+) versus Par1b (-/-) brains. a, Schematic representation of the cytokine array kit. PC, positive controls, NC, negative controls. **b,** Representative blots from P5 Par1b (+/+) and Par1b (-/-) brains showing levels of 40 known cytokines. **c,** Quantification revealed no significant changes greater than 1.2 fold, n=2, *p<0.05.

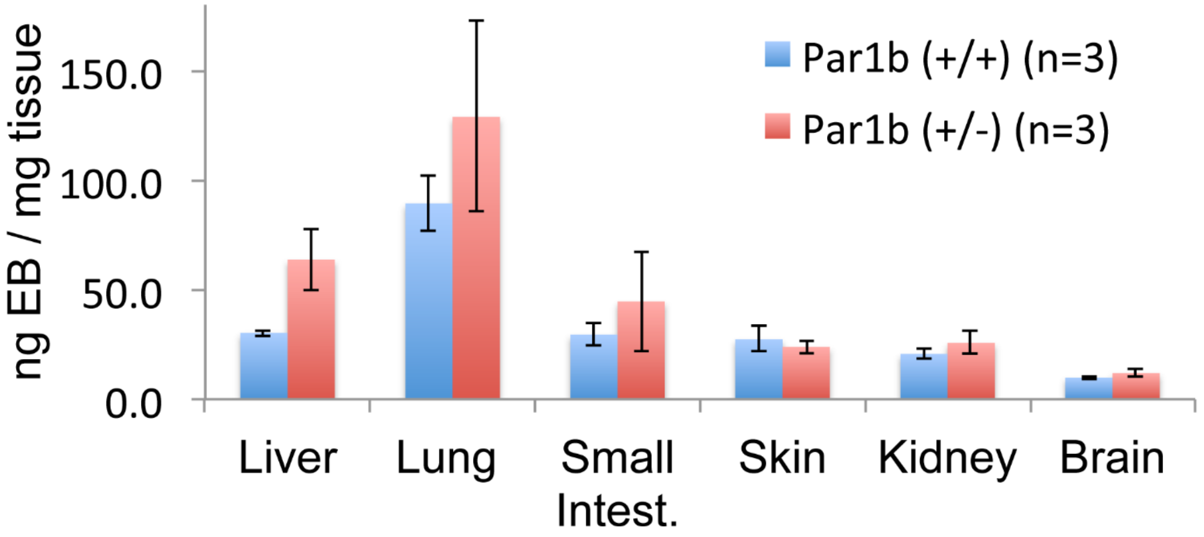

Figure S7. No significant change in extravasation of Evan Blue dye was found in different organs from Par1b (+/+) versus (+/-) mice. 30 minutes after a tail vein injection of Evans Blue dye in both Par1b (+/+) versus Par1b (+/-) mice, mice were sacrificed and different organs were collected and weighed. Evans Blue dye was extracted from tissue with formamide. Concentration of ng of Evans Blue dye per mg of tissue was measured, n=3. No significant difference was found between Par1b (+/+) and (+/-) mice in the brain or other organs collected, suggesting an intact blood-brain barrier and normal blood vessel permeability in general.

TABLE S1a Two-Way ANOVA MEASURE OF PRIMARY MICROGLIA CIRCULARITY

shRAN Tranfection - Treatment	n-microglia	Mean	SEM	df factor (shRNA or treatment)	df factor (shRNA or treatment)	df (within groups)	F	p	Sig.	Post hoc Bonferroni	Post hoc Sig.
**Lucif-shRNA Ctrl**	472	0.313	0.007	shRNA	1	2015	89	< 0.0001	***	LC vs LA < 0.0001	***
**Lucif-shRNA ATP**	542	0.422	0.009	Treatments	1	2015	108.9	< 0.0001	***	LC vs PC < 0.0001	***
**Par1b-shRNA Ctrl**	522	0.414	0.008	shRNA versus Treatment	1	2015	5.944	0.015	*	LC vs PA < 0.0001	***
**Par1b-shRNA ATP**	483	0.482	0.010							LA vs PC > 0.9999	ns
										LA vs PA < 0.0001	***
										PC vs PA < 0.0001	***

TABLE S1b One-Way ANOVA PRIMARY MICROLGIA ENGULFMENT OF NEURONAL PARTICLES

shRNA Transfection	n-microglia	Mean	SEM	df (between groups)	df (within groups)	F	p	Sig.	Post hoc Bonferroni	Post hoc Sig.
**Lucif-shRNA Ctrl**	37	4.568	0.659	1	69	12.39735	0.001	***	0.001	***
**Par1b-shRNA Ctrl**	34	8.412	0.890							

TABLE S2 One-Way ANOVA MICROGLIA DENSITY DEVELOPMENTALLY

Postnatal Day - Genotype	n-mice	n-images	Mean	SEM	df (between groups)	df (within groups)	F	p	Sig.	Post hoc Bonferroni	Post hoc Sig.
**P5 (+/+)**	6	38	0.871	0.021	1	78	24.536	< 0.0001	***	< 0.0001	***
**P5 (+/-)**	7	42	0.402	0.097							
**P9 (+/+)**	7	30	2.779	0.080	1	72	7.352	0.008	**	0.008	**
**P9 (+/-)**	3	44	2.234	0.214							
**P30 (+/+)**	5	34	2.511	0.072	1	68	3.777	0.056		0.056	**ns**
**P30 (+/-)**	5	36	2.249	0.116							
**P120 (+/+)**	7	26	2.244	0.078	1	62	10.085	0.002	**	0.002	**
**P120 (+/-)**	5	38	1.681	0.182							

TABLE S3a Type III Test of Fixed Effects/Linear Mixed Model MICROGLIA DEVELOPMENTAL MORPHOLOGY - BRANCH COMPLEXITY

Morphology-Complexity	Postnatal Day	n-mice	n-microglia	Mean	Stand Dev	SEM	Coeff Var	Numerator df	Denominator df	F	Type III Test of Fixed Effects	Sig.
Primary 10 WT	P5	4	40	21.18	9.274	1.466	43.80%	1	74	5.166	0.026	*
Primary 10 Het	P5	4	36	15.842	7.306	1.218	46.10%					
Primary 15 WT	P5	4	40	15.628	10.204	1.613	65.30%	1	74	7.183	0.009	**
Primary 15 Het	P5	4	36	11.442	8.53	1.422	74.50%					

Morphology-Complexity	Postnatal Day	n-mice	n-microglia	Mean	Stand Dev	SEM	Coeff Var	Numerator df	Denominator df	F	Type III Test of Fixed Effects	Sig.
Secondary 25 WT	P5	4	40	10.325	10.336	1.634	100.10%	1	2761.421	5.244	0.022	*
Secondary 25 Het	P5	4	36	4.825	5.901	0.984	122.30%					

Morphology-Complexity	Postnatal Day	n-mice	n-microglia	Mean	Stand Dev	SEM	Coeff Var	Numerator df	Denominator df	F	Type III Test of Fixed Effects	Sig.
Tertiary WT												ns
Tertiary Het												ns

Morphology-Complexity	Postnatal Day	n-mice	n-microglia	Mean	Stand Dev	SEM	Coeff Var	Numerator df	Denominator df	F	Type III Test of Fixed Effects	Sig.
Quart 15 WT	P120	5	37	18.108	12.617	2.074	0.458	1	72	7.096	0.01	**
Quart 15 Het	P120	4	37	10.93	10.358	1.703	52.60%					

TABLE 3b Type III Test of Fixed Effects/Linear Mixed Model MICROGLIA DEVELOPMENTAL MORPHOLOGY - STRUCTURAL CHARACTERISTICS

Morphology-Intersections	Postnatal Day	n-mice	n-microglia	Mean	Stand Dev	SEM	Coeff Var	Numerator df	Denominator df	F	Type III Test of Fixed Effects	Sig.
Intersections WT												ns
Intersections Het												ns

Morphology-Avg Branch Leng	Postnatal Day	n-mice	n-microglia	Mean	Stand Dev	SEM	Coeff Var	Numerator df	Denominator df	F	Type III Test of Fixed Effects	Sig.
Avg Branch Leng 25 WT	P120	5	37	97.657	28.047	4.611	28.70%	1	5.755	6.504	0.045	*
Avg Branch Leng 25 Het	P120	3	37	77.551	25.948	4.266	36.70%					

Morphology-Nodes	Postnatal Day	n-mice	n-microglia	Mean	Stand Dev	SEM	Coeff Var	Numerator df	Denominator df	F	Type III Test of Fixed Effects	Sig.
Nodes 20 WT	P5	4	40	2.35	1.889	0.299	68.30%	1	137.229	6.992	0.009	**
Nodes 20 Het	P5	4	36	3.472	2.372	0.395	80.40%					
Nodes 20 WT	P30	5	40	10.15	4.029	0.637	39.70%	1	4.433	16.68	0.012	*
Nodes 20 Het	P30	4	39	8.821	3.41	0.546	38.70%					
Nodes 25 WT	P30	5	40	6.75	3.349	0.53	49.60%	1	6.97	6.543	0.038	*
Nodes 25 Het	P30	4	39	5.513	2.955	0.473	53.60%					
Nodes 25 WT	P120	5	37	7.081	3.148	0.518	44.50%	1	1563.744	4.576	0.033	*
Nodes 25 Het	P120	3	37	5.054	2.624	0.431	51.90%					

Morphology-Endings	Postnatal Day	n-mice	n-microglia	Mean	Stand Dev	SEM	Coeff Var	Numerator df	Denominator df	F	Type III Test of Fixed Effects	Sig.
Endings 20 WT	P5	4	40	3.475	1.935	0.306	55.70%	1	2111.169	6.784	0.009	**
Endings 20 Het	P5	4	36	4.611	3.174	0.529	68.80%					
Endings 25 WT	P120	5	37	11.757	4.058	0.667	34.50%	1	472.307	4.12	0.043	*
Endings 25 Het	P120	4	37	8.757	2.976	0.489	34.00%					

TABLE S4 One-Way ANOVA EARLY MICROLGIA ENGULFMENT OF NEURONAL PARTICLES

Genotype	n-mice	n-microglia	Mean	SEM	df (between groups)	df (within groups)	F	p	Sig.	Post hoc Bonferroni	Post hoc Sig.
**P5 (+/+)**	6	39	56.578	4.781	2	114	3.680	0.028	**	KO vs WT 0.025	*
**P5 (+/-)**	7	44	71.505	6.231						Het vs WT 0.296	**ns**
**P5 (-/-)**	2	34	82.272	8.555						Het vs KO 0.749	**ns**

TABLE S5 One-Way ANOVA MICROGLIA DYNAMICS

Genotype	n-mice	n-microglia	Mean	SEM	df (between groups)	df (within groups)	F	p	Sig.	Post hoc Bonferroni	Post hoc Sig.
**Par1b (+/+)**	6	60	177.735	3.667	1	128	58.029	< 0.0001	***	< 0.0001	***
**Par1b (+/-)**	7	70	146.482	1.351							

TABLE S6a Two-Way ANOVA MICROGLIA DENSITY POST-TBI

Genotype-Surgery	n-mice	n-images	Mean	Stand Dev	SEM	df factor (geno or surgery)	df factor (geno or surgery)	df (within groups)	F	p	Sig.	Post hoc Bonferroni	Post hoc Sig.
**WT-Naïve**	5	48	0.019	0.011	0.002	Genotype	1	240	5.279	0.022	*	WT-N vs WT-S > 0.9999	**ns**
**WT-Sham**	3	36	0.024	0.014	0.002	Surgery	2	240	44.03	< 0.0001	***	WT-T vs WT-S < 0.0001	***
**WT-CCI-TBI**	6	51	0.070	0.047	0.007	Genotype x Surgery	2	240	1.452	0.022	*	WT-T vs WT-N < 0.0001	***
**Het-Naïve**	3	27	0.021	0.009	0.002							Het-N vs Het-S 0.038	*
**Het-Sham**	3	27	0.047	0.024	0.005							Het-T vs Het-S 0.001	***
**Het-CCI-TBI**	6	57	0.082	0.060	0.008							Het-T vs Het-N < 0.0001	***
												WT-N vs Het-N > 0.9999	**ns**
												WT-S vs Het-S 0.065	**ns**
												WT-T vs Het-T 0.361	**ns**

TABLE S6b Two-Way ANOVA OVERALL FLUORESCENT INTENSITY POST-TBI

Genotype-Surgery	n-mice	n-images	Mean	Stand Dev	SEM	df factor (geno or surgery)	df factor (geno or surgery)	df (within groups)	F	p	Sig.	Post hoc Bonferroni	Post hoc Sig.
**WT-Naïve**	5	48	14.555	2.51	0.36	Genotype	1	240	8.451	0.004	***	WT-N vs WT-S > 0.9999	**ns**
**WT-Sham**	3	36	12.991	6.636	1.11	Surgery	2	240	20.14	< 0.0001	***	WT-T vs WT-S < 0.0001	***
**WT-CCI-TBI**	6	51	19.82	8.887	1.24	Genotype x Surgery	2	240	5.329	0.005	**	WT-T vs WT-N 0.002	**
**Het-Naïve**	3	27	13.292	5.321	1.02							Het-N vs Het-S 0.006	**
**Het-Sham**	3	27	19.619	6.176	1.19							Het-T vs Het-S 0.136	**ns**
**Het-CCI-TBI**	6	57	23.112	10.162	1.35							Het-T vs Het-N < 0.0001	***
												WT-N vs Het-N 0.481	**ns**
												WT-S vs Het-S 0.001	***
												WT-T vs Het-T 0.022	*

TABLE S7a One-Way ANOVA APOPTOSIS ASSAY

Genotype	Tissue	n-mice	Mean	df (between groups)	df (within groups)	F	p	Sig.	Post hoc Bonferroni	Post hoc Bonferroni	Post hoc Sig.
**Par1b (+/+)**	Cortex (RSG)	5	0.216	2	10	0.338	0.721	**ns**	Het vs KO	0.287	**ns**
**Par1b (+/-)**		5	0.256						Het vs WT	0.57	**ns**
**Par1b (-/-)**		3	0.28						KO vs WT	0.781	**ns**
**Par1b (+/+)**	Cortex (V1)	5	0.246	2	10	3.82	0.059	**ns**	Het vs KO	0.105	**ns**
**Par1b (+/-)**		5	0.242						Het vs WT	1	**ns**
**Par1b (-/-)**		3	0.141						KO vs WT	0.088	**ns**
**Par1b (+/+)**	HIP (CA1a)	5	0.348	2	10	0.063	0.939	**ns**	Het vs KO	0.258	**ns**
**Par1b (+/-)**		5	0.362						Het vs WT	0.333	**ns**
**Par1b (-/-)**		3	0.349						KO vs WT	0.031	*
**Par1b (+/+)**	HIP (CA3)	5	0.353	2	10	0.402	0.679	**ns**	Het vs KO	0.243	**ns**
**Par1b (+/-)**		5	0.404						Het vs WT	0.673	**ns**
**Par1b (-/-)**		3	0.425						KO vs WT	0.826	**ns**

TABLE S7b One-Way ANOVA BLOOD VESSEL PERMEABILITY ASSAY

Genotype	Tissue	n-mice	Mean	df (within groups)	F	p	Sig.	Post hoc Bonferroni	Post hoc Sig.
**Par1b (+/+)**	**Liver**	3	30.31201	4	5.48103	0.079	**ns**	0.079	**ns**
**Par1b (+/-)**		3	63.67437						
**Par1b (+/+)**	**Lung**	3	128.84899	4	0.76411	0.431	**ns**	0.431	**ns**
**Par1b (+/-)**		3	89.38995						
**Par1b (+/+)**	**Small Intest**	3	44.60626	4	0.41037	0.557	**ns**	0.557	**ns**
**Par1b (+/-)**		3	29.48445						
**Par1b (+/+)**	**Skin**	3	23.87226	4	0.27984	0.625	**ns**	0.625	**ns**
**Par1b (+/-)**		3	27.38443						
**Par1b (+/+)**	**Kidney**	3	25.73061	4	0.73834	0.439	**ns**	0.439	**ns**
**Par1b (+/-)**		3	20.68881						
**Par1b (+/+)**	**Brain**	3	11.93685	4	0.96318	0.382	**ns**	0.382	**ns**
**Par1b (+/-)**		3	9.92105						
